# Exploring the linkage between health technology assessment and decision making during COVID-19 public health emergency in a developing country: analysis of processes and results

**DOI:** 10.1017/S0266462324000473

**Published:** 2024-11-04

**Authors:** Santiago Hasdeu, Anabel Beliera, Jorgelina Alvarez, Julián Sánchez Viamonte

**Affiliations:** 1 RedArets (Red Argentina Pública de Evaluación de Tecnologías Sanitarias), Neuquén, Argentina; 2 Instituto Patagónico de Humanidades y Ciencias Sociales (IPEHCS, CONICET/UNComa), Neuquen, Argentina; 3 Universidad Nacional de Cuyo, Mendoza, Argentina; 4 Facultad de Medicina Universidad Nacional de Mar del Plata, Mar del Plata, Argentina

**Keywords:** health policy, decision making, organizational, coronavirus infections, technology assessment, biomedical, developing countries

## Abstract

**Objectives:**

The decision-making (DM) process in public administration is the subject of research from different perspectives and disciplines. Evidence-based policies, such as health technology assessment (HTA), are not the only support on which public policies are designed. During the COVID-19 pandemic WHO, national and subnational institutions developed HTA reports to guide DM. Despite this, inadequate variability was observed in the health technologies recommended and reimbursed by different provincial Health Ministries in a federally organized developing country like Argentina. The processes and results of DM on health technologies for COVID-19 in Health Ministries of Argentina were inquired.

**Methods:**

A retrospective research design was developed, with triangulation of quantitative and qualitative methods. We retrieved information for the years 2020–2021 through document review of official webpages, surveys, and interviews with decision-makers of the 25 Argentinian Ministries of Health. We analyzed the recommendations and reimbursement policies of seven health technologies.

**Results:**

In contradiction with WHO’s policies, ivermectine, inhaled ibuprofen, convalescent plasma and equine serum were widely recommended by most of Argentina’s health ministries outside a clinical trial context, with risks for patients and a huge opportunity cost.

**Conclusions:**

Despite an important HTA institutional capacity, the impact of HTA organizations and their technical reports was limited. Health Ministries with institutionalized HTA units had more adherence to WHO recommendations, but the influence of different technical and political criteria was identified. Power relations within and outside the administration, the pharmaceutical industry and academics, the media, social pressure, the judicial and legislative powers, and the political context strongly influenced DM.

## Introduction

The health emergency due to the COVID-19 pandemic, as a unique and disruptive event, involved profound changes and transformations that affected public administration, organizations, communities, and the entire spectrum of human life (1). In the health care sector, this impact was especially important: COVID-19 caused high rates of morbidity and mortality, saturation of health care systems, and a global economic crisis. These effects made COVID-19 a particular challenge for developing countries (2). The great number of health technologies proposed for COVID-19 treatment, without scientific evidence of efficacy, generated great tensions that decision-makers had to face (3). The large amount of information published in different scientific journals was estimated at hundreds of thousands, with different levels of quality and, in many cases with, contradictory information, fake news, and fraud (4). For example, because of fraud on the hydroxychloroquine study, The Lancet journal issued an expression of concern to alert readers and had to retract a published article (5), and WHO temporarily interrupted the hydroxychloroquine arm of the SOLIDARITY trial until confirmation of fake information and then decided to continue it (6). WHO’s department of communication implemented measures to fight infodemics and misinformation (7). To illustrate the need for reliable information on health technology, the Cochrane Collaboration’s systematic review of ivermectin for COVID-19 was the most consulted systematic review of all time (8). Although the authors were uncertain about its efficacy and safety, it was extensively used outside the context of clinical trials in various Latin American countries (9). Similar situations were described across the region with other health technologies.

Health Technology Assessment (HTA), “a multidisciplinary process that uses explicit methods to determine the value of a health technology in different points in their life cycle,” is a key tool in supporting decision making on the selection of health technologies (10). Consequently, the World Health Organization (WHO) promotes the establishment of an institutional framework for HTA-based decision-making to stimulate institutional responsibility and create links between the use of HTA in the decision-making process (11).

However, some authors have noted a weak linkage between HTA and DM in Latinamerica (11) and in Argentina (12).

Feinstein observed that not all policies are evidence-based (13), March & Olsen (2011) proposed the “garbage can model” and suggested that public administration can be an example of what they consider “organized anarchies,” where there is ambiguity in the objectives (which are poorly defined by the actors), in the technology to be used, and in the participation of the actors (which tends to vary over time) (14). Decisions end up arising from the chance encounter of conjunctural factors, among problems, solutions, participants, and opportunities.

In public administration, the decision-making process can be heavily influenced by technical and political criteria, the experience, profile, and values of the decision-makers, the characteristics of the advisors, power relations within and outside of the administration, the media, social pressures, and the political and economic context (15–17).

In the healthcare epidemiological context, the uncertainty regarding benefits, risks, and cost–benefit balance of interventions, makes decision-making even more complex than in other areas of public administration (18). Consequently, there are many technical, organizational, and managerial reasons that make it necessary to evaluate policies (19).

Argentina is a developing country with 24 provinces. Due to its federal organization, the nation’s health ministry guides and suggests actions, but each provincial health ministry has autonomy to make its own health decisions.

HTA in Argentina comprises a National HTA Commission (CONETEC) (20), a national public HTA network (RedArets)(22) and HTA units in six provinces, several universities, and other public and private institutions (12). These publicly funded organizations receive evaluation requests from their governments, industry, and citizens, conduct prioritization processes, and openly publish their reports (20). Recommendations from CONETEC and RedArets are not legally binding, so each health subsector makes its own coverage and reimbursement decisions. This lack of legislation supporting HTA could limit its impact (11;12).

Courts play a particularly prominent role in healthcare decision-making in Argentina and the rest of Latin America. Most countries have a constitutional court that acts as a safety net for fundamental rights. Patients can appear in court to gain access to a service that is guaranteed in the public or private plan but delivered inadequately, or to gain access to a service or technology not included in the coverage (21).

Very little has been published on the evaluation of public health policies during the pandemic, especially on health technologies recommended and used for COVID-19. The few studies published focused on non-pharmacological interventions, such as lockdown policies, (23). Therefore, the objective of this study was to explore the processes and results of decision-making and its linkage with health technology assessment during the COVID-19 pandemic in a federally organized developing country.

## Methods

The unit of analysis was the health ministries of Argentina, including the National Health Ministry and the 24 provincial ministries. The analysis population included decision-makers and advisors of these ministries. The period of analysis was the two year period from 2020 to 2021. The investigation focused on decision-making processes and results regarding the following health technologies: ivermectin, convalescent plasma, equine serum, inhaled ibuprofen, remdesivir, tocilizumab, and dexamethasone. These health technologies were selected because they represented a heterogeneous set of controversial and non-controversial interventions; some were recommended by the WHO and some were not. They also included a mix of high- and low-cost medicines, nationally manufactured and imported products, patent-protected and generic technologies and medicines, and derivatives of human and equine plasma.

Data were collected on the following aspects:

- The health technologies recommended and reimbursed by the different health ministries of Argentina during the pandemic;
- The actors involved in decision-making within each ministry
- The health technology assessment units involved; their conformation and technical recommendations
- Internal tensions and the degree of influence of actors external to health ministries.
- Laws and bills on COVID-19 medicines and court rulings forcing the coverage of medicines for COVID-19
- Guidelines and technical reports on the seven health technologies mentioned above, published by international and national organizations and academic societies

## Data collection

Data collection was organized in different stages:

First, a revision of the official web pages of the national and 24 provincial health ministries, RedArets (National Public Network of HTA of Argentina) and the national scientific societies related to internal medicine, infectious disease, respiratory medicine, and intensive care was conducted. During the pandemic, the websites of the 25 health ministries were an important mechanism for disseminating protocols of care, epidemiological reports, and recommendations for personal protection to medical teams. The revision included:

- The technical reports, practice guidelines, and statements related to health technologies for COVID-19 published between 1 January 2020 and 31 December 2021 were selected and analyzed.
- To explore the international reference guidelines and HTA reports related to COVID-19 treatment, searches were made in the official web pages of WHO (24) and PAHO (25).
- National and provincial laws and bills related to the subject were collected through a search on specialized legal websites of Argentina (http://leg.msal.gov.ar).
- Individual and collective lawsuits related to the selected health technologies in Federal and Provincial Courts were searched in specialized legal websites of Argentina (http://www.saij.gob.ar/home, http://www.infojus.com )
- A review of the principal national digital media, (www.clarin.com, www.lanacion.com.ar, www.pagina12.com.ar, www.cronica.com.ar) was also conducted using the selected technologies as keywords. This source of information complemented the previously mentioned sources in order to identify health technology recommendations and reimbursement policies, laws, and lawsuits in each of the Argentine provinces.

The second stage of data collection consisted of a semi-structured survey (see Supplementary File List of official webpages of Ministries of Health) aimed at gathering general information about the advisory teams for decision-making, the participation of internal and external influencers, and the final results in terms of recommended, financed, not recommended, and non-financed technologies. The construction of the survey followed the sequential steps of domain and item generation based on bibliographic review and discussion with experts on survey methodology, item reduction, validity and reliability evaluation, pilot test, adjustments, dissemination, and analysis. It was sent on May 2022, in electronic format (Google-form) to key actors related to decision-making on health technologies in the national and provincial health ministries. Snowball sampling was used to identify potential participants. Invitations to participate were sent to the 25 health ministries directly and indirectly, through the collaboration of scientific-academic societies, networks, and health professional unions. The priority was to garner representation from as many provinces as possible and to include individuals who had been in advisory positions or members of the emergency or health technology evaluation committees of each ministry. There was no prior selection of relevant positions, given that those who formed the emergency committees and gave technical recommendations varied greatly among health ministries.

Based on the aforementioned stages, information was collected on the health policy followed by each of the health ministries for each of the seven selected technologies. We sought to see if each ministry followed or not the recommendations of official HTA organizations. The policy of each ministry was compared with the recommendations of WHO, PAHO, CONETEC, and RedArets, since these organizations were in consensus regarding the seven selected technologies. It was also noted which health ministries had institutionalized HTA areas or units. For each health ministry, the average adherence to the official HTA reports was expressed as a percentage, taking the 7 health technologies as 100 percent. For each of the selected health technologies, the average adherence of health ministries was shown as a percentage. Traffic light colors were used to illustrate the adherence of the health ministries to the recommendations of official HTA agencies. When a health ministry followed the official HTA recommendation for a given technology, the box was painted green; when it disagreed, the box was painted red. When within a health ministry there were different technical recommendations or contradictions between internal technical recommendations and the issued policy, the box was painted yellow.

The third stage consisted of in-depth interviews (range from 45 to 60 min) with a sample of 8 selected key actors identified in previous stages. The domains of the interview script were developed based on the results of the surveys. We explored experiential aspects and feelings about the decision-making process, as well as delved deeper into the decision-making process, looking for cases to illustrate the functioning of the “black box” of the decision-making processes in public policy (26). Interviews were carried out in person or via an audiovisual platform (Zoom®), requesting authorization for their recording, with subsequent transcription and analysis. Two interviewers conducted the interviews between November and December 2022, keeping a record of non-verbal language. It was significant to record the anguish (even tears) of several technical staff of the ministries when they reported that their recommendations were sometimes not followed, knowing that this could potentially imply greater risk for patients or hospital workers. Also, the stress when they recalled the feeling of urgency and pressure to make recommendations in a very short time, with scarce evidence and a high death toll.

## Results

The results of public policies related to the seven selected health technologies in the National and Provincial Health Ministries of Argentina are summarized in Table 1.

**Table 1.**
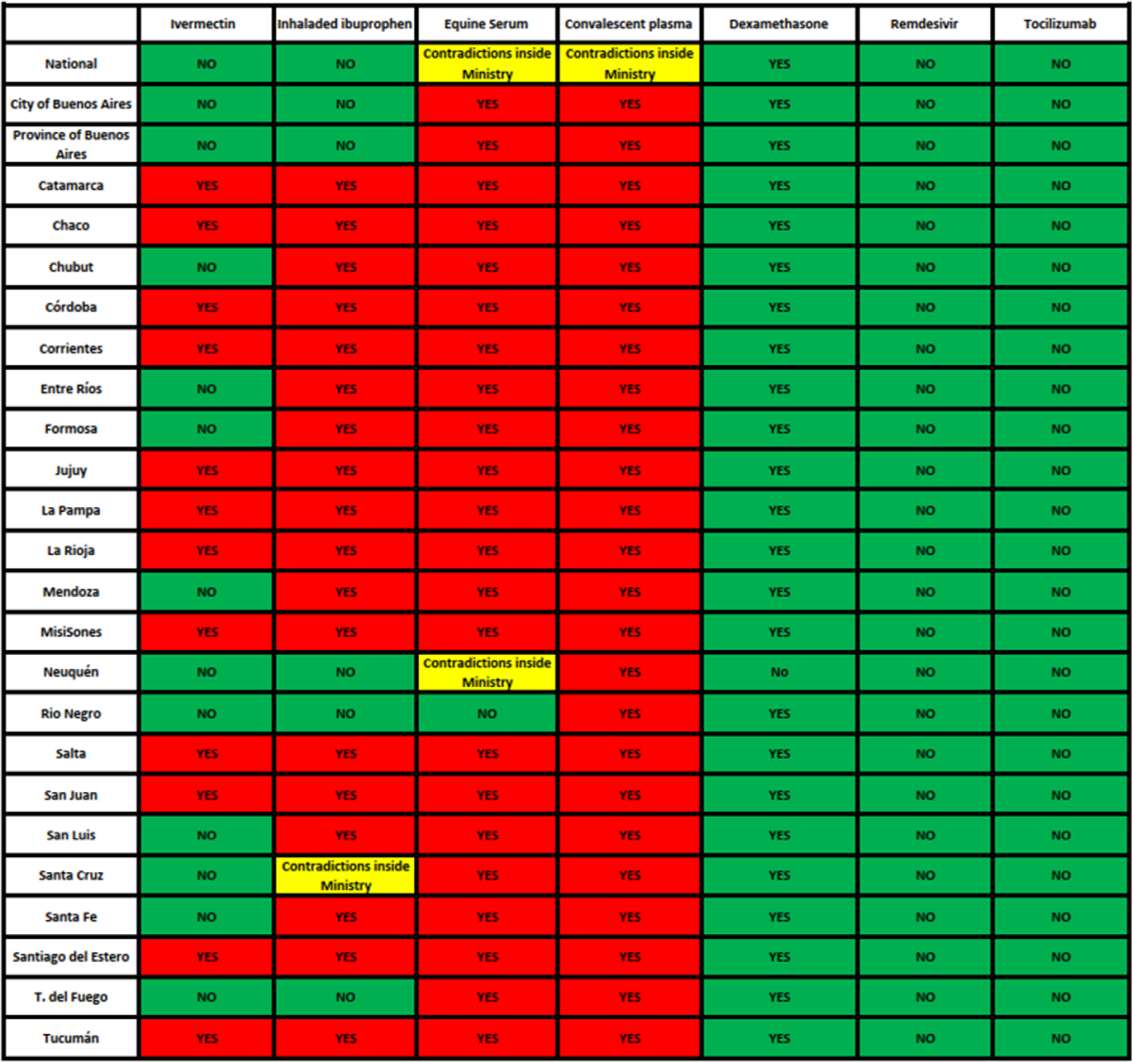
Adherence to official HTA recommendations issued by WHO, PAHO, CONETEC and RedArets by health ministries and by selected health technologies In Argentina

*Source:* Based on results of surveys, interviews and web page analysis.

As can be seen in Table 1, there was no Health Ministry with complete adherence to official HTA recommendations for the seven selected health technologies in Argentina. The Rio Negro Health Ministry showed the highest adherence (86 percent), followed by the National Ministry of Health and the Provinces of Buenos Aires, Neuquén, Tierra del Fuego, and the City of Buenos Aires (71 percent of adherence).

Among the selected health technologies, the adherence was 100 percent for dexamethasone, remdesivir, and tocilizuamb. All of the Ministries recommended and supplied convalescent plasma outside clinical trial contexts. 96 percent of health ministries recommended and provided equine serum, 77 percent recommended and provided inhaled ibuprofen, and 50 percent did so with ivermectine.

Among the health ministries, seven had institutionalized HTA Units (National, City of Buenos Aires, and provinces of Buenos Aires, Mendoza, Santa Fe, Neuquén and Rio Negro) and 18 did not. Overall the adherence to official WHO and PAHO recommendations was higher in ministries with institutionalized HTA units (Table 2), particularly in relation to ivermectin (100 percent versus 39 percent adherence), inhaled ibuprophen (71 percent versus 5 percent adherence) and equine serum (14 percent versus 0 percent adherence). There were no differences for the implemented policies in relation to convalescent plasma, dexamethasone, remdesivir, and tocilizumab.

**Table 2.** Adherence to official WHO recommendations during 2020–2021 by health ministries with and without HTA Units in Argentina (Percentage)

Ministry of Health/Health Technology	Ivermectin	Inhaled ibuprofen	Equine serum	Convalescent plasma	Dexamethasone	Remdesivir	Tocilizumab
Ministries of Health with HTA Units (*n* = 7)	100	71	14	0	100	100	100
Ministries of Health without HTA Units (*n* = 18)	39	5	0	0	100	100	100

*Source:* Based on results of surveys, interviews and web page analysis.

In the qualitative analysis, the surveys and interviews showed complementary information about the processes that led to these results. The surveys obtained 34 responses, belonging to 14 different health ministries. All of the country’s regions (NorthEast, NorthWest, Cuyo, Centre and Patagonia) were represented among the respondents. Because of the snowball sampling, we can not estimate the response rate. We did not identify differences between the health ministries from which we obtained answers and those from which we did not. Among the respondents, 90 percent previously worked in health ministries and 10 percent worked in hospitals and were specifically called to conform committees or advisory teams for the management of the pandemic in a health ministry. Their profiles included specialists in public health, hemotherapy, infectious diseases, intensive care, internal medicine, epidemiology, bioethics, immunology, family medicine, and pharmacists with experience in research and health technology assessment. In some ministries, nurses, biochemists, and economists also participated in the advisory teams. At least one or more members of the seven ministerial HTA units answered the surveys and interviews.

The results indicate that it was predominantly the health ministers themselves who made the decisions on which health technologies were recommended and reimbursed, but in some cases the participants stated that decisions came from other areas. For example, in many provinces, the parliament enacted laws to guarantee the access to ivermectin or inhaled ibuprofen. In some cases, decisions “came directly from the central government, from a higher level.” There were also some decisions that were ordered by the judiciary. These were mainly on an individual basis, where a judge made it compulsory to provide inhaled Ibuprofen to a hospitalized patient, despite the disagreement of his treating physicians (27). Documents were retrieved, where individual patients and/or civil society organizations sued the executive power (provincial or national) through judicial channels, demanding coverage of health technologies for COVID-19 treatment that was neither recommended nor covered by the public health care institutions. In some cases, for example in Buenos Aires city, judges granted these individual demands, forcing the executive branch to cover medicines or treatments that contradicted the treatment policies issued by WHO and local health authorities (27). Some respondents stated that their Minister of Health returned from meetings with the national and other provincial health ministers with a decision made on health technology and ordered to buy it immediately. This reveals the leadership and influence of the national ministry over provincial ministries, at least in some situations. There did not appear to be any instances where the ministers voted on a decision. Respondents mentioned multiple meetings to analyze and discuss health technologies for which there was no evidence of effectiveness but which had significant public interest and media support. Respondents mentioned that in some cases “experts” from other provinces and even from other countries were asked to provide advice on health technologies. In most of the cases, official HTA reports were an input for decision-making. In some cases, the health ministers publicly stated that their HTA units would make the decision, although this was not the norm.

Some comments from respondents indicated that decision making was not only guided by technical rationality but also considered multiple processes and relationships in which different actors were involved.

“The work with experts was spasmodic due to the need of the authorities, … there was no work in periodic assessments; many times we worked without knowing the topic to be dealt with.” (Epidemiologist and advisor).

“…. It is good to clarify that because of the place that I had to occupy during the pandemic, I had to see that many of the decisions (the majority) were made from a political and not a health point of view.” (Infectologist and advisor).

“The pressure from the media was very strong.” (Decision Maker).

Many respondents made similar comments, which suggests that the media was very influential in the decision-making process during the pandemic in Argentina. When asked specifically about one technology, respondents from different health ministries, commented about equine serum:

“I believe that despair in the face of so many serious and fatal cases led the authorities to make political decisions regarding the use, for example, of Equine serum.” (Intensive care therapist and advisor).

“Equine serum was a ministerial decision. I do not know what technical endorsement the decision was made on.” (Pharmacist and advisor).

Thus, some of the decisions of health authorities were not evidence-based, and in some cases, fear guided or strongly influenced some of the decisions made by the health authorities.

## Discussion

Due to its federal organization, each jurisdiction in Argentina defined its own public policies on health technologies for COVID-19. The results show an important heterogeneity in the decisions about which technologies were recommended and reimbursed by each health ministry. Despite having official HTA reports and guidelines from WHO, PAHO, CONETEC, and RedArets, which showed consistency between them (see Supplementary File Table of Recommendations), the degree of adherence of the health ministries was relatively low. Ivermectin, inhaled ibuprofen, convalescent plasma, and equine serum were widely recommended and reimbursed, contrary to official HTA recommendations. Health ministries with institutionalized HTA units showed more adherence to WHO’s policies, so institutionalized HTA could have been a facilitator of evidence-based decision-making. Nonetheless, all health ministries recommended and provided convalescent plasma outside of clinical research settings, and most ministries purchased and distributed equine serum. These policies promoted the extensive use of ineffective and inappropriate technologies, put the health of the population at risk, and had an enormous opportunity cost. In some of these cases, the pharmaceutical industry and some scientific societies and organizations openly supported the use of health technologies in contradiction with official HTA recommendations. For example, the Infectious disease society of Argentina (SADI) and other national scientific societies openly supported the use of equine serum and convalescent plasma (28). The reason for this is unclear.

The study identified multiple factors influencing the decision-making processes. These included the different conformations of the advisory teams, power struggles within health ministries, political pressures, opinions of other provincial ministers, laws and the judicialization of health, the media, and the opinions of academics, who often had contradictory views on some technologies for COVID-19. Public opinion was also a strong influence in pressing for access to pharmaceuticals that had significant promotion in the media. Within some ministries of health, contradictions were due to different opinions among various areas or work teams from the same institution. These disputes for “the truth” in the academic field were evidenced in contradictory scientific publications about the efficacy of some of the selected health technologies.

The advisors and decision-makers were questioned in their values and convictions during the pandemic and directly pressured by the media, which exacerbated their fears and contradictions. Issues related to power, the role of central government, science and academics, and the influence of social networks and the media, among others, came into tension for some of these individuals.

Very little research has been published regarding public health policy decision-making in pandemics. A study in Brazil endorsed non-pharmacological interventions (23), suggesting an influence of political parties on subnational lockdown policies during the pandemic.

The current study showed that the decision-making process in the different ministries of health of Argentina was highly heterogeneous, and that social media, other ministries, law, judiciary, academics, and public organizations had an important influence on this process in many instances.

Subirats (15) stated that evidence-based decision-making is rare because of the decisional complexity of collective problems and the fickleness of the contexts in which decisions are made, and there are often many problems occurring simultaneously with a high degree of uncertainty and conflict. This was seen in the current study, where the complex interplay of internal and external factors and influential actors had profound effects on the veracity and integrity of the decision-making process within the health ministries in the pandemic context, which was characterized by unusually high pressure and scrutiny from the media, social networks, and the public.

In many cases, the respondents stated that they were unaware of the intimate mechanisms by which the authorities made some of the decisions about technologies. According to Easton, these decisions remained within the “black box of decision-making in public administration” (26).

The potential limitations of this study are related to its retrospective design, which may be subject to various biases; these include selection bias in the context of the snowball sampling and information bias related to the interviewer and the recall bias. However, combining qualitative and quantitative approaches to data collection allowed the triangulation of results from complementary sources.

The results of this study indicate that it would be desirable to increase the link between HTA technical teams and decision makers in the ministries of health of Argentina to reduce the number of decisions made without scientific support. The literacy of decision-makers regarding HTA and evidence-based medicine was not assessed in this study which could provide additional information. It is necessary to develop future research on the decision-making process for health interventions that are made under normal and abnormal circumstances. The evaluation of the health and economic consequences of public policies should always be conducted, and the use of HTA should be encouraged.

## Supporting information

Hasdeu et al. supplementary material 1 Hasdeu et al. supplementary material

Hasdeu et al. supplementary material 2 Hasdeu et al. supplementary material

Hasdeu et al. supplementary material 3 Hasdeu et al. supplementary material

## Supplementary material

The supplementary material for this article can be found at http://doi.org/10.1017/S0266462324000473.
