# Supplementary material for: Exploring the linkage between health technology assessment and decision making during COVID-19 public health emergency in a developing country: analysis of processes and results
Source: Int J Technol Assess Health Care. 2024 Nov 4;40(1):e42. doi: 10.1017/S0266462324000473 (PMC11563179; doi:10.1017/S0266462324000473)
Supplement: Hasdeu et al. supplementary material 1 — Hasdeu et al. supplementary material [file S0266462324000473sup001.docx]

Supplementary File

List of Health Ministries of Argentina and their official webpages included in the electronic search:

National Ministry of Health:

<https://www.argentina.gob.ar/salud/conetec/publicaciones-ets>

Buenos Aires Province Ministry of Health: <https://www.gba.gob.ar/salud>

Ciudad Autónoma de Buenos Aires Ministry of Health: <https://www.buenosaires.gob.ar/salud>

Catamarca Province Ministry of Health: <http://www.catamarcasalud.gob.ar/>

Chaco Province Ministry of Health: <https://www.saludchaco.gob.ar/>

Chubut Province Ministry of Health: <http://www.chubut.gov.ar/sitio/salud/>

Córdoba Province Ministry of Health: <https://www.cba.gov.ar/salud/>

Corrientes Province Ministry of Health: <https://www.mscorrientes.gob.ar/>

Entre Ríos Province Ministry of Health: <https://www.entrerios.gov.ar/msalud/>

Formosa Province Ministry of Health: <https://www.formosa.gob.ar/sitio/humanoysocial>

Jujuy Province Ministry of Health: <https://salud.jujuy.gob.ar/>

La Pampa Province Ministry of Health: <https://www.salud.lapampa.gob.ar/>

La Rioja Province Ministry of Health: <http://www.larioja.gov.ar/msalud/>

Mendoza Province Ministry of Health: <http://www.mendoza.gov.ar/salud/>

Misiones Province Ministry of Health: <https://www.salud.misiones.gob.ar/>

Neuquén Province Ministry of Health: <https://www.saludneuquen.gob.ar/>

Río Negro Province Ministry of Health: <https://www.salud.rionegro.gov.ar/>

Salta Province Ministry of Health: <https://www.salud.salud.salta.gov.ar/>

San Juan Province Ministry of Health: <https://www.salud.sanjuan.gob.ar/>

San Luis Province Ministry of Health: <https://www.sanluis.gov.ar/ms/>

Santa Cruz Province Ministry of Health: <http://salud.santacruz.gov.ar/>

Santa Fe Province Ministry of Health: <https://www.santafe.gob.ar/ms/>

Santiago del Estero Province Ministry of Health: <http://www.sde.gov.ar/ms/>

Tierra del Fuego Province Ministry of Health: <https://www.saludtdf.gob.ar/>

Tucumán Province Ministry of Health: <https://www.saludtuc.gob.ar/>
