# Supplementary material for: Exploring the linkage between health technology assessment and decision making during COVID-19 public health emergency in a developing country: analysis of processes and results
Source: Int J Technol Assess Health Care. 2024 Nov 4;40(1):e42. doi: 10.1017/S0266462324000473 (PMC11563179; doi:10.1017/S0266462324000473)
Supplement: Hasdeu et al. supplementary material 2 — Hasdeu et al. supplementary material [file S0266462324000473sup002.docx]

Supplementary file Survey on decision-making on health technologies for COVID-19:

Informed Consent

My name is Santiago Hasdeu, I am a researcher at the National University of Comahue and I coordinate a research team made up of health and social science professionals from the Argentina provinces of Neuquén, Buenos Aires and Mendoza.

We are inviting you to participate as a respondent in a national research on the decision-making process in the context of the COVID 19 pandemic, which is called “Evaluation of processes and results in decision-making about health technologies in the context of the COVID-19 pandemic.” This survey can be completed in approximately 10 minutes. In particular, we will ask you questions related to the following health technologies: Inhaled Ibuprofen, Ivermectin, Equine Serum, Convalescent Plasma, Remdesivir, Tocilizumab and Dexamethasone.

The present study was evaluated by the Bioethics and Research Commission on Human Beings of the Province of Neuquén, and has been financed by the National Ministry of Health of Argentina (Salud Investiga Scholarship program).

Your participation is voluntary. We ask that you take the time necessary to analyze whether you wish to participate considering what is best for you and that you consult with people you trust if you wish. Analyze the following information and ask anything you consider necessary to the email of the main researcher hasdeusanti@gmail.com and/or to the email of the Neuquén Bioethics Commission caibsh_nqn@yahoo.com.ar.

All the information that we will consult with you will be considered confidential and exclusively covers the period between January 2020 and December 2021.

1-Do you agree to participate in the study by answering this survey?

YES/NO

2-During the 2020-21 period did you work in any of the following Health Ministries?

National Ministry of Health

Buenos Aires Province Ministry of Health

Ciudad Autónoma de Buenos Aires Ministry of Health

Catamarca Province Ministry of Health

Chaco Province Ministry of Health

Chubut Province Ministry of Health

Córdoba Province Ministry of Health

Corrientes Province Ministry of Health

Entre Ríos Province Ministry of Health

Formosa Province Ministry of Health

Jujuy Province Ministry of Health

La Pampa Province Ministry of Health

La Rioja Province Ministry of Health

Mendoza Province Ministry of Health

Misiones Province Ministry of Health

Neuquén Province Ministry of Health

Río Negro Province Ministry of Health

Salta Province Ministry of Health

San Juan Province Ministry of Health

San Luis Province Ministry of Health

Santa Cruz Province Ministry of Health

Santa Fe Province Ministry of Health

Santiago del Estero Province Ministry of Health

Tierra del Fuego Province Ministry of Health

Tucumán Province Ministry of Health

3-If in the previous question you answered that you work/worked in more than one Ministry of Health, the following questions will focus on the one you consider your main place of work. Please mention which one it is.

4-Please describe your position/s in said Ministry:

5-What is your main place of work?

- Ministry of Health

- Charitable work

- National University

- Scientific Society

- Means of communication

- Hospital

- Another

6-In the Ministry of Health where you work/ed during pandemic, was there a technical team formed that has made decisions or advised on the medications and technologies to be used for COVID-19?

Yes/No

7-If you answered yes in the previous question, please describe the name of said technical team

8-Could you mention the actors (positions, professional profiles and names) of those who were part of said technical team at some point during the 2020-2021 period (you may include those actors who have been part temporarily)?

9-Did you participate in any of the meetings where the coverage/reimbursement or non-coverage/non reimbursment of any of the health technologies mentioned in the introduction was discussed and/or decided?

Yes/No

10-If the previous question was answered affirmatively, who do you consider to have been key actors in the discussion processes on each of these technologies in the MoH? (please mention positions, professional profiles and names)

11-Did the MoH where you work/ed explicitly recommended the use of any of the following treatments for COVID 19 during the 2020-2021 period?

|  | Yes | No | Don´t know |
| --- | --- | --- | --- |
| Inhaled Ibuprofen |  |  |  |
| Ivermectin |  |  |  |
| Equine Serum |  |  |  |
| Convalescent Plasma |  |  |  |
| Remdesivir |  |  |  |
| Tocilizumab |  |  |  |
| Dexamethasone |  |  |  |

12-The MoH where you work/ed, purchased and/or delivered to the health care centers any of the following treatments for COVID 19 during the 2020-2021 period?

|  | Yes | No | Don´t know |
| --- | --- | --- | --- |
| Inhaled Ibuprofen |  |  |  |
| Ivermectin |  |  |  |
| Equine Serum |  |  |  |
| Convalescent Plasma |  |  |  |
| Remdesivir |  |  |  |
| Tocilizumab |  |  |  |
| Dexamethasone |  |  |  |

13-In your Jurisdiction was there any law that established the use of any of the mentioned health technologies?

Yes/No

14-If you answered affirmatively to the previous question, please describe for which health technology said law was, and provide information.

15-When making decisions about health technologies, were the recommendations of any of the following scientific/health institutions and organizations taken into account during the 2020-2021 period?

|  | WHO | PAHO | CONETEC | RedArets |
| --- | --- | --- | --- | --- |
| Inhaled Ibuprofen | Yes/No/Don´t know | Yes/No/Don´t know | Yes/No/Don´t know | Yes/No/Don´t know |
| Ivermectin | Yes/No/Don´t know | Yes/No/Don´t know | Yes/No/Don´t know | Yes/No/Don´t know |
| Equine Serum | Yes/No/Don´t know | Yes/No/Don´t know | Yes/No/Don´t know | Yes/No/Don´t know |
| Convalescent Plasma | Yes/No/Don´t know | Yes/No/Don´t know | Yes/No/Don´t know | Yes/No/Don´t know |
| Remdesivir | Yes/No/Don´t know | Yes/No/Don´t know | Yes/No/Don´t know | Yes/No/Don´t know |
| Tocilizumab | Yes/No/Don´t know | Yes/No/Don´t know | Yes/No/Don´t know | Yes/No/Don´t know |
| Dexamethasone | Yes/No/Don´t know | Yes/No/Don´t know | Yes/No/Don´t know | Yes/No/Don´t know |

16-If you have answered YES in any of the options in the previous question, you are invited to provide details (Ex: The report from Society X that recommended using/not using drug Z was considered in our MoH)

17-For decision making, were the recommendations of any of the following actors external to the MoH taken into consideration?

|  | NGOs | Amparo appeal for protection or judicial measure | Media | Other/s |
| --- | --- | --- | --- | --- |
| Inhaled Ibuprofen | Yes/No/Don´t know | Yes/No/Don´t know | Yes/No/Don´t know | Yes/No/Don´t know |
| Ivermectin | Yes/No/Don´t know | Yes/No/Don´t know | Yes/No/Don´t know | Yes/No/Don´t know |
| Equine Serum | Yes/No/Don´t know | Yes/No/Don´t know | Yes/No/Don´t know | Yes/No/Don´t know |
| Convalescent Plasma | Yes/No/Don´t know | Yes/No/Don´t know | Yes/No/Don´t know | Yes/No/Don´t know |
| Remdesivir | Yes/No/Don´t know | Yes/No/Don´t know | Yes/No/Don´t know | Yes/No/Don´t know |
| Tocilizumab | Yes/No/Don´t know | Yes/No/Don´t know | Yes/No/Don´t know | Yes/No/Don´t know |
| Dexamethasone | Yes/No/Don´t know | Yes/No/Don´t know | Yes/No/Don´t know | Yes/No/Don´t know |

18-If you checked "other/s" in the previous question, please describe.

19-Information about the respondent (We remind you of our commitment to maintain confidentiality about all the information collected)

20-Email

21-Age

22-Gender

23-Seniority in position (in years)

24-Seniority in the Ministry of Health (in years)

25-What is your Profession?

26-If you wish, you can add below any comments that you consider important.

Thank you very much
