# Supplementary material for: Exploring the linkage between health technology assessment and decision making during COVID-19 public health emergency in a developing country: analysis of processes and results
Source: Int J Technol Assess Health Care. 2024 Nov 4;40(1):e42. doi: 10.1017/S0266462324000473 (PMC11563179; doi:10.1017/S0266462324000473)
Supplement: Hasdeu et al. supplementary material 3 — Hasdeu et al. supplementary material [file S0266462324000473sup003.docx]

Supplementary file

Table of recommendations on health technologies for COVID-19 by selected organizations:

|  | World Health Organization | PanAmerican Health Organization | National Commission on Health Technology Assessment of Argentina (CONETEC) | National Public Network of Health Technology Assessment of Argentina (RedArets) |
| --- | --- | --- | --- | --- |
| Inhaled Ibuprofen | Not recommended | Not recommended | Not recommended | Not recommended |
| Ivermectin | Not recommended | Not recommended | Not recommended | Not recommended |
| Equine Serum | Not recommended | Not recommended | Not recommended | Not recommended |
| Convalescent Plasma | Not recommended | Not recommended | Not recommended | Not recommended |
| Remdesivir * | Not recommended | Not recommended | Not recommended | Not recommended |
| Tocilizumab * | Not recommended | Not recommended | Not recommended | Not recommended |
| Dexamethasone # | Recommended | Recommended | Recommended | Recommended |

* Recommendations for Remdesivir and Tocilizumab were negative (not recommended for COVID-19) during the 2020-2021 period and this recommendation changed in 2022

# Recommendations for Dexamethasone were negative (not recommended) until September 2020
